# Metabolism of Tanshinone IIA, Cryptotanshinone and Tanshinone I from Radix Salvia Miltiorrhiza in Zebrafish

**DOI:** 10.3390/molecules17078617

**Published:** 2012-07-18

**Authors:** Yingjie Wei, Ping Li, Changmei Wang, Yunru Peng, Luan Shu, Xiaobin Jia, Wenquan Ma, Bing Wang

**Affiliations:** 1 Key Laboratory of New Drug Delivery System of Chinese Materia Medica, Jiangsu Provincial Academy of Chinese Medicine, 100 Shizi Street, Nanjing 210028, China; Email: wyj970@163.com (Y.W.); hanxi186@yahoo.com.cn (C.W.); pengyunru@yahoo.com.cn (Y.P.); shuluan2006@hotmail.com (L.S.); 2 State Key Laboratory of Natural Medicines and Department of Pharmacognosy, China Pharmaceutical University, 24 Tongjia Lane, Nanjing 210009, China; 3 Biology & Medicine Science and Technology Industry Park of Weifang Hi-Tech Development Zone, Crossroads of Jiankang East Street and Gaoxin Road 2, Weifang 261061, China;Email: mwq3119@126.com (W.M.); wb1078@163.com (B.W.)

**Keywords:** zebrafish, phase I metabolism, tanshinone IIA, cryptotanshinone, tanshinone I, Radix Salvia miltiorrhiza

## Abstract

The study aimed to investigate the potential of zebrafish in imitating mammal phase I metabolism of natural compounds. Three diterpenoid quinones from Radix Salvia miltiorrhiza, namely tanshinone IIA (TIIA), cryptotanshinone (Cry) and tanshinone I (TI) were selected as model compounds, and their metabolites mediated by zebrafish were characterized using a high-performance liquid chromatography coupled ion-trap mass spectrometry (HPLC/IT-MSn) method with electrospray ionization in positive mode. The separation was performed with a Zorbax C-18 column using a binary gradient elution of 0.05% formic acid acetonitrile/0.05% formic acid water. According to the MS spectra and after comparison with reference standards and literature reports, hydroxylation, dehydrogenation or D-ring hydrolysis metabolites of TIIA and Cry but not of TI were characterized, which coincided with those reported using regular *in vivo* or *in vitro* metabolic analysis methods, thus verifying that zebrafish can successfully imitate mammalian phase I metabolism which instills further confidence in using zebrafish as a novel and prospective metabolism model.

## 1. Introduction

The zebrafish (*Danio rerio*), a well-characterized model vertebrate in the fields of molecular genetics and developmental biology, is becoming an increasingly popular pre-clinical testing model organism in drug toxicology and screening for its similar genes and complex organs found in mammals, with a view to functioning as an *in vivo* screen to reduce the number of compounds and to save cost of mammals in drug development [1,2,3,4,5,6]. However, knowledge about drug metabolism in zebrafish is relatively limited. Xenobiotic metabolism has substantial implications for disease modelling, ecotoxicology, toxicity testing and drug screening, due to the potential for the metabolic activation and deactivation of compounds. To strengthen the justification for the use of zebrafish in drug discovery and toxicity testing, it is essential that the status of xenobiotic metabolism be understood in this model species throughout development. 

It has reported that zebrafish expressed metabolic enzymes such as cytochrome P450 isoforms or conjugation enzymes (GST, UGTs and SULTs) similar to mammals’ [7,8,9,10,11,12], in addition, many studies including those from our laboratory, have demonstrated that a variety of fish species, especially zebrafish, possess similar types of metabolic capability as that observed in mammalian species [13,14,15,16,17,18,19,20,21,22,23,24,25]. For example, step-wise deglycosylation and hydroxylation metabolites of notoginsenoside R_1_, ginsenoside Rg_1_ and ginsenoside Rb_1_, and deglycosylation metabolites of icariin, epimedin A and epimedin C by adult zebrafish were found [23,25]. Calycosin metabolites formed from glucuronidation, glucosylation, sulfation, oxidation or a combination of two of these metabolisms by zebrafish larvae were also identified [19]. These results suggested that drug metabolism using zebrafish can reflect integrated results of mammalian methods, whereas single *in vitro* methods could not make these possible, which shed a light on adopting zebrafish as a whole-organism model for examining drug metabolism with obvious advantages of low cost, trace amounts of compound needed, easy set up and high efficiency. 

It is believed that active or toxic components *in vivo* include not only parent components but also their metabolites; therefore, metabolic study is of great importance in Traditional Chinese Medicines (TCMs). However, there are still limitations for metabolic study methods so far using either *in vivo* or *in vitro* models in TCM research, and it is important to provide more choice for metabolism study of TCMs using zebrafish [23,25,26]. 

**Figure 1:**
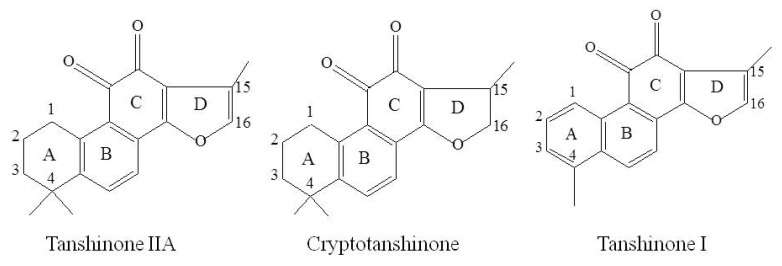
Structures of TIIA, Cry and TI in Radix Salvia miltiorrhiza used in this study.

*Salvia miltiorrhiza*, a well-known Chinese herb named Dan-Shen, is used for treatment of various kinds of diseases, especially for cardiovascular ones [27,28]. TIIA, Cry and TI (Figure 1) are three major bioactive diterpenoid quinones, which had been extensively investigated pharmacokinetically and metabolically [29,30,31,32,33,34,35,36,37,38]. It is believed that tanshinones mainly undergo phase I type metabolic transformations such as hydroxylation and dehydrogenation, which were dependent on the degree of saturation and the substituent groups on the skeleton. Dehydrogenation was the major metabolic modification for cryptotanshinone with saturated A and D rings, and hydroxylation was the major metabolic pathway for tanshinone IIA with a saturated A ring, while as for tanshinone I, bearing unsaturated A and D rings simultaneously, no metabolites were detected [34].

Our previous study reported that zebrafish can successfully imitate mammal intestinal bacteria in deglycosylation of saponins and flavonoids from the Chinese herbs named Sanqi and Yingyanghuo, respectively [23,25]. It is generally known that phase I metabolism mediated by cytochrome P450 enzymes is also one of important drug metabolic pathways, so more efforts are needed in exploring such potential in the zebrafish model. As a part of the continuing efforts to this end, this paper selected TIIA, Cry and TI, to see if zebrafish can imitate the known phase I metabolism of natural components. In addition to previous reports using zebrafish larvae [19,39], our results provide updated and valuable evidence for the first time using adult zebrafish in a phase I metabolic study of trace components of TCMs, which instills further confidence in making zebrafish a prospective metabolism model.

## 2. Results and Discussion

### 2.1. Analysis of Metabolic Components of TIIA, Cry and TI after Zebrafish Exposure by HPLC/IT-MSn

In comparison with the blank samples, metabolites of TIIA, Cry and TI were characterized after zebrafish exposure. These compounds and their metabolites exhibited their quasi-molecular ions [M+H]^+^ or [M+Na]^+^ in positive mode, and were ascertained by a general analysis of the mass spectra or fragmentation behaviors, retention times and comparing with reference data and some of standards. The major product ions of the metabolites, along with their HPLC retention times are summarized in Table 1. 

**Figure 2:**
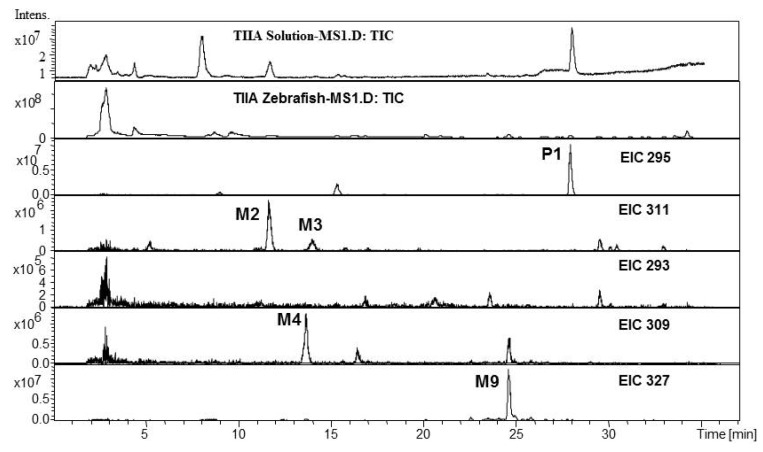
Total ion chromatogram and extracted ion chromatograms for TIIA after exposure to zebrafish for 24 h.

*Metabolites of TIIA in Zebrafish*: Parent component TIIA (P1, MW 294) and its monohydroxylated, dihydroxylated and dehydrogenated metabolites were identified in either solution matrix or zebrafish homogenate (Figure 2). M2 and M3 all gave the quasi-molecular ions of [M+H]^+^ 311 and [M+Na]^+^ 333, and the MW was deduced as 310, 16 Da higher than that of TIIA, hence they were identified as monohydroxyl TIIA, which were probably 3α-hydroxytanshinone II A and przewaquinone A (MW 310) according to previous reports [29,34]. M4 (MW 308) gave the quasi-molecular [M+H]^+^ ion of *m/z* 309, 2 Da less than that of the monohydroxylated metabolite of TIIA, hence it was identified as a dehydrogenated product of hydroxyl TIIA.

**Table 1:** MS data for TIIA, Cry, TI and their metabolites after zebrafish exposure for 24 h.

Compounds	Retention Time (min)	Quasi-Molecular Ions Peak		HPLC/ESI-MSn Or fragment Ions	MW	Metabolite presumed	TIIA group	Cry group	TI group	Current Metabolism Reference
		[M+H]^+^	[M+Na]^+^							
P1	27.7	295.2	317.1	MS2[295]: 277.1[M+H-H_2_O]^+^, 249.1[M+H-H_2_O-CO]^+^	294.2	Tanshinone IIA	+	+		[ 29,32,34,38]
				MS3[295→277]: 262.1[M+H-CH_3_]^+^, 249.1[M+H-H_2_O-CO]^+^, 231.1[M+H-2H_2_O-CO]^+^, 221.1[M+H-H_2_O-2CO]^+^, 206.1[M+H-H_2_O-2CO-CH_3_]^+^						
				MS4[295→277→249]: 234[M+H-H_2_O-CO-CH_3_]^+^, 221.1,206.2, 191.2[M+H-H_2_O-2CO-2CH_3_]^+^						
P2	22.8	297.2	319.2	MS2[297]: 297.2[M+H]^+^, 279.2[M+H-H_2_O]^+^, 264.1[M+H-H_2_O-CH_3_]^+^, 251.1[M+H-H_2_O-CO]^+^, 237.1[M+H-CO-CH_3_OH]^+^, 209.1[M+H-2CO-CH_3_OH]^+^	296.2	Cryptotanshinone		+		[ 34,38]
				MS3[297→279]: 264.1,251.1, 237.1,209.1						
P3	23.4	277.1	299.1	MS2[277]: 259.1[M+H-H_2_O]^+^, 249.1[M+H-CO]^+^, 231.1[M+H-2H_2_O-CO]^+^, 221.1[M+H-H_2_O-2CO]^+^	276.1	Tanshinone I			+	[ 34]
				MS3[277→249]: 234.1[M+H-CO-CH_3_]^+^, 231.1[M+H-CO-H_2_O]^+^, 221.1[M+H-2CO]^+^, 203[M+H-2CO-H_2_O]^+^, 193.1[M+H-3CO]^+^, 178[M+H-3CO-CH_3_]^+^						
M1	10.9	311.2	333.2		310.2	Tanshinone IIB		+		[ 29,32,34,38]
M2	11.6	311.2	333.2	MS2[311]: 293.1[M+H-H_2_O]^+^, 275.1[M+H-2H_2_O]^+^, 263.1[M+H-H_2_O-2CH_3_]^+^, 251.1[M+H-CO-CH_3_OH]^+^, 235.1[M+H-H_2_O-2CH_3_-CO]^+^	310.2	3α-Hydroxytanshinone IIA	+	+		[ 29,32,34,38]
				MS3[311→293]: 275.1[M+H-2H_2_O]^+^, 265.3[M+H-CO-H_2_O]^+^, 251.1[M+H-CO-CH_3_OH]^+^, 247.2 [M+H-CO-2H_2_O]^+^, 229.1[M+H-CO-3H_2_O]^+^, 219.1[M+H-2CO-2H_2_O]^+^						
M3	13.9	311.2	333.3		310.2	Przewaquinone A	+			[ 29,32,34,38]
M4	13.6	309.1		MS2[309]: 291.1[M+H-H_2_O]^+^, 277.2[M+H-CH_3_OH]^+^, 265.2[M+H-2CH_3_-CH_3_OH+H_2_O]^+^, 247.0[M+H-2CH_3_-CH_3_OH]^+^, 235.2[M+H-4CH_3_-CH_3_OH+H_2_O]^+^	308.1	Dehydrogenated product ofthe hydroxylated metaboliteof tanshinone IIA	+			[ 34,38]
				MS3[309→291]: 291.2[M+H-H_2_O]^+^, 273.2[M+H-2H_2_O]^+^, 261.2[M+H-H_2_O-2CH_3_]^+^, 245.1[M+H-2H_2_O-CO]^+^						
				MS3[309→281]: 263.1,253.1[M+H-2CO]^+^, 239.1[M+H-2CO-CH_3_OH+H_2_O]^+^, 211.0[M+H-3CO-CH_3_OH+H_2_O]^+^, 201.2[M+H-4CO-CH_3_OH+2H_2_O]^+^, 183.0[M+H-4CO-CH_3_OH+H_2_O]^+^						
M5	34.0	313.3	335.3	256.3[M+H-CH_3_-CH_3_OH-CO+H_2_O]^+^, 230.3[M+H-CH_3_-CH_3_OH-2H_2_O]^+^,	312.3	Hydroxyl cryptotanshinone		+		[ 34,38]
M6	19.1	315.3	337.3	300.3[M+H-CH_3_]^+^, 286.2[M+H-CH_3_-CH_3_OH+H_2_O]^+^, 270.3[M+H-3CH_3_]^+^	314.3	Tanshinone V		+		[ 34,38]
M7	28.5	315.3	337.3	300.1[M+H-CH_3_]^+^, 282.3[M+H-CH_3_-H_2_O]^+^, 262.3[M+H-2CO-CH_3_+H_2_O]^+^,	314.3	Tanshinone V isomer		+		[ 34,38]
M8	25.9	327.3	349.3	309.3[M+H-H_2_O]^+^, 299.2[M+H-CO]^+^, 277.2[M+H-H_2_O-CH_3_OH]^+^	326.3	Dihydroxyl tanshinone IIA		+		[ 32]
M9	24.6	327.2	349.2	309.2[M+H-H2O]^+^, 299.2[M+H-CO]^+^	326.2	Dihydroxyl tanshinone IIA	+	+		[ 32]
M10	29.2	329.3	351.2	311.3[M+H-H_2_O]^+^, 301.2[M+H-CO]^+^	328.3	Dihydroxyl cryptotanshinone		+		[ 33]
M11	30.1	329.3	351.3	311.3[M+H-H_2_O]^+^, 299.2[M+H-2CH_3_]^+^, 293.2[M+H-2H_2_O]^+^, 261.2[M+H-2H_2_O-CH_3_OH]^+^, 237.2[M+H-2H_2_O-2CO]^+^, 219.2[M+H-3H_2_O-2CO]^+^, 199.1[M+H-2H_2_O-3CO]^+^	328.3	Dihydroxyl cryptotanshinone		+		[ 34]
M12	32.8	329.3	351.2	311.3[M+H-H_2_O]^+^, 286.2[M+H-CO-CH_3_]^+^	328.3	Dihydroxyl cryptotanshinone		+		[ 34]

+ Detected.

M9 gave quasi-molecular ions of [M+H]^+^ 327 and [M+Na]^+^ 349, and MW was deduced as 326, 32 Da higher than that of TIIA, and a major fragment [M+H-18]^+^ ion at *m/z* 309, formed via loss of H_2_O, indicating that it was probably dihydroxylated metabolite of TIIA.

**Figure 3:**
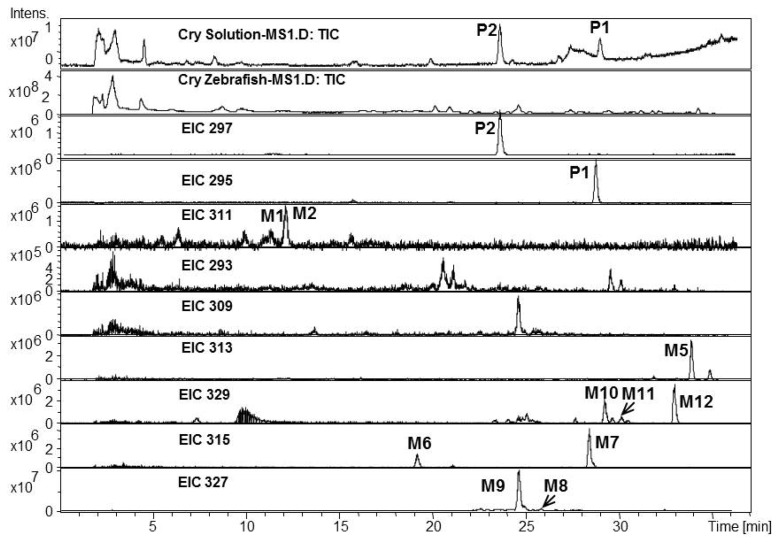
Total ion chromatogram and extracted ion chromatograms for Cry after exposure to zebrafish for 24 h.

*Metabolites of Cry in Zebrafish:* Parent component Cry (P2, MW 296) and its dehydrogenated, monohydroxylated, dihydroxylated and D-ring-opened metabolites were identified in either solution matrix and zebrafish homogenate (Figure 3). Tanshinone IIA (P1, MW 294) was the major dehydrogenated metabolite of Cry by comparing with standard and MS spectra.

M1, M2 (MW 310), corresponding to a 16 Da shift from that of TIIA, were monohydroxylated metabolites of TIIA; M8, M9 (MW 326), with a 32 Da shift from that of TIIA, were dihydroxytanshinone IIA; M5 gave the quasi-molecular ion of [M+H]^+^ 313, 16 Da higher than that of Cry, hence it was identified as monohydroxycryptotanshinone; M10, M11 and M12 all gave the quasi-molecular ion of [M+H]^+^ 329, 32 Da higher than that of Cry, and major fragment [M+H-18]^+^ ions at *m/z* 311, formed via loss of H_2_O, hence they were identified as dihydroxylated metabolites of Cry; M6, M7 gave the quasi-molecular ion of [M+H]^+^ 315, 18 Da higher than that of Cry, and were thus identified as a D-ring-opened metabolite named tanshinone V and its isomer, according to the literature [34,38].

**Figure 4:**
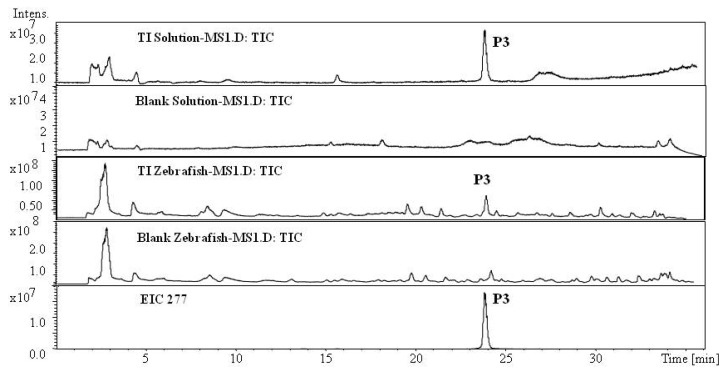
Total ion chromatogram and extracted ion chromatograms for TI after exposure to zebrafish for 24 h.

*Metabolites of TI in Zebrafish*: No metabolite of TI in zebrafish was found, except for the parent component TI (P3, MW 276), which was identified from a standard and its MS spectra (Figure 4), this result was also consistent with previous report of rat metabolism. 

**Figure 5:**
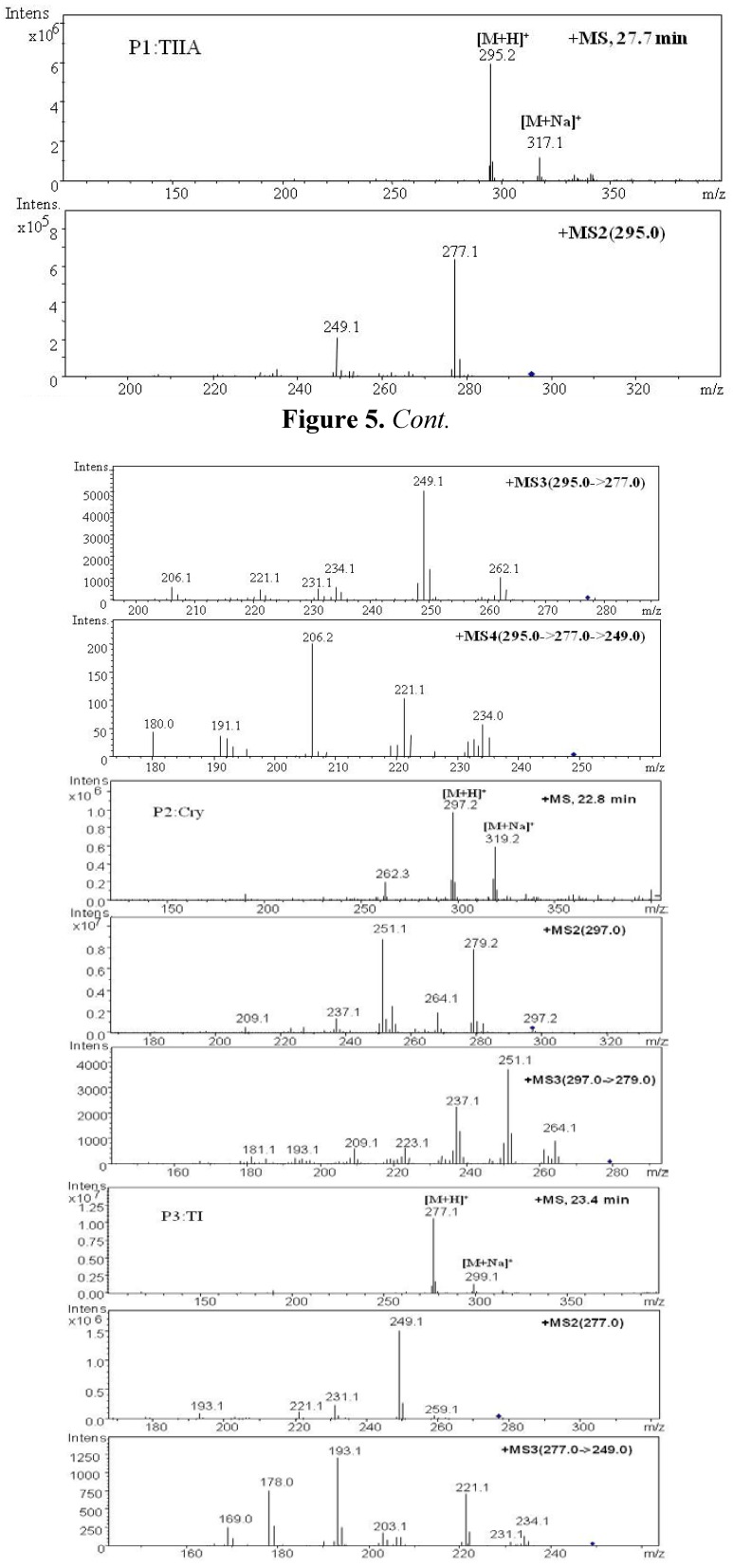
Positive ion ESI mass spectra and MSn spectra of parent components of TIIA, Cry and TI.

**Figure 6:**
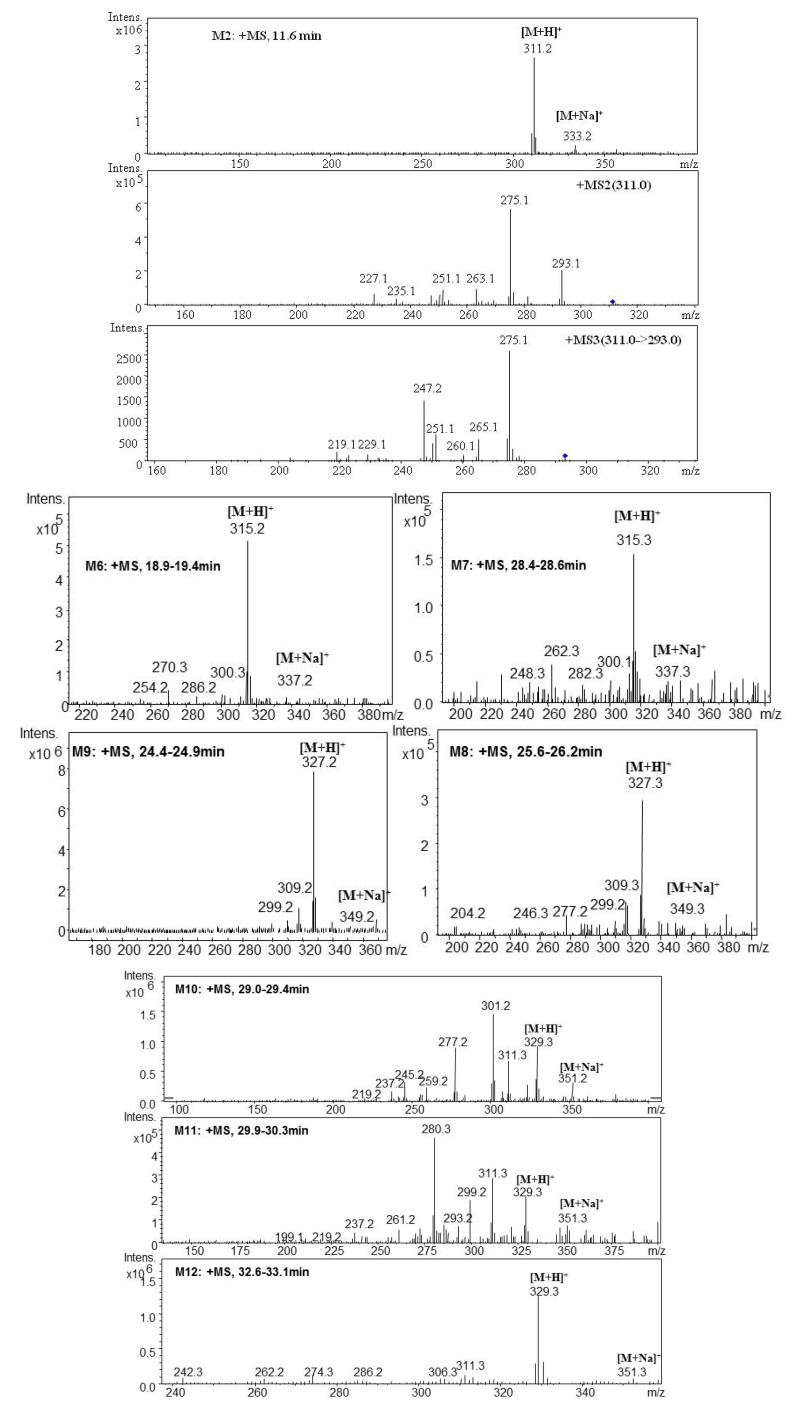
Positive ion ESI mass spectra and MSn spectra of metabolites components of TIIA and Cry by zebrafish.

The representative MS spectra of parent components and metabolites are shown in Figure 5 and Figure 6.

### 2.2. Rationality and Advantages of Metabolic Study with Zebrafish Compared to Existing Models

Our study verified that zebrafish can successfully imitate regular phase I metabolism in elucidating the mechanism of metabolism of TIIA, Cry and TI from Radix Salvia miltiorrhiza. It is believed that tanshinones mainly undergo phase I type of metabolic reactions such as hydroxylation and dehydrogenation *in vitro*, and different metabolic reactions for tanshinones were dependent on the degree of saturation and the substituent group in the skeleton [34]. 

**Figure 7:**
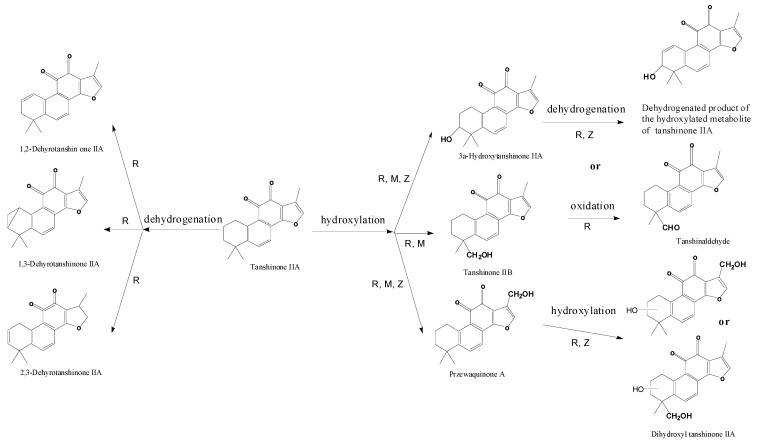
Comparison of metabolic pathways of TIIA between zebrafish and the current metabolism methods. Note: R represents Rat, M stands for Rat liver microsomes and Z for zebrafish.

Our results indicated that hydroxylation was the major metabolic mechanism of TIIA by zebrafish, which was highly consistent with the existing metabolism results (metabolic pathways are shown in Figure 7). Earlier several publications on metabolism of TIIA were complementary or similar though not completely consistent with each other, but the common and major metabolic pathway of TIIA of *in vitro* (rat liver microsomes) [29] and *in vivo* (rat) [34,38] metabolism was hydroxylation, and dehydrogenation is a secondary pathway. In our present metabolism study of TIIA in zebrafish, two monohydroxyl TIIA (MW 310) and one dihydroxyl TIIA (MW 326) were found, which underwent the major similar metabolic transformations as TIIA in rat, dehydrogenated TIIA (MW 292) was not found, the reason may be due to its hydroxylation into a dehydrogenated product of hydroxyl TIIA (MW 308). Thus it can be seen that hydroxylation rather than dehydrogenation was the major metabolic pathway of TIIA possessing a saturated A ring, which is consistent with the conclusions of [34].

**Figure 8:**
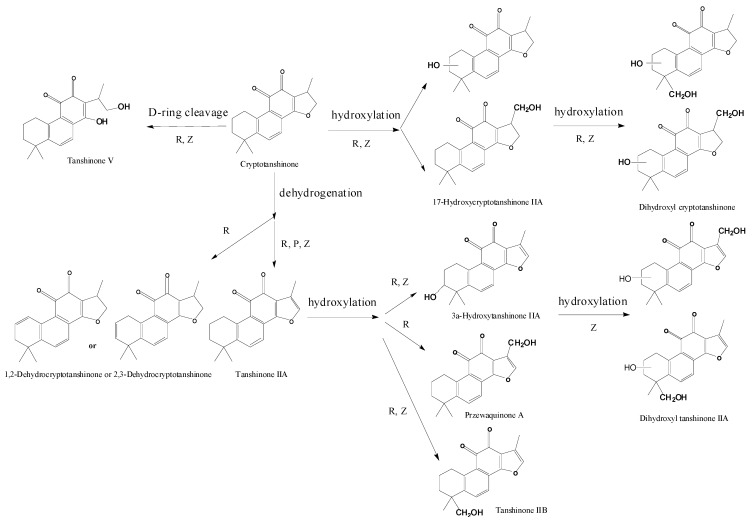
Comparison of metabolic pathways of Cry between zebrafish and the current metabolism data. R: Rat; P: Pig and Z: Zebrafish.

Our results also showed that metabolic mechanisms of Cry by zebrafish were hydroxylation and dehydrogenation, which were also highly consistent with the existing metabolism results (the metabolic pathways are shown in Figure 8). Cry had been found to be rapidly metabolized into dehydrogenated metabolite tanshinone IIA to a great extent when administered to mammals such as pig and rat [34,37,38], which has been widely reported in previous publications. In addition, several trace hydroxylated metabolites were also characterized, such as monohydroxylated metabolites of TIIA (MW 310), monohydroxylated metabolites of Cry (MW 312), dihydroxylated metabolites of Cry (MW 328) and D-ring-opened metabolite tanshinone V (MW 314) [34,38]. In our present metabolism study of Cry in zebrafish, dehydrogenated metabolite tanshinone IIA (MW294), two monohydroxyl tanshinone IIA (MW 310), monohydroxyl cryptotanshinone (MW 312), three dihydroxyl cryptotanshinone (MW 328) and two D-ring-opened metabolite (MW 315) were found, in addition, two isomers of dihydroxyl tanshinone IIA (MW 326) were also reported for the first time. It can be seen that Cry underwent the highly similar metabolic reactions in zebrafish and in rat.

Except for the parent component TI, no other transformed metabolite was found, which was consistent with the results obtained from *in vivo* study in rat or *in vitro* study with rat liver microsomes [34]. In the present metabolic study of Cry, TIIA and TI using zebrafish, only trace amounts (about 1–2 mg) of compounds are needed, which were far less than that required for rat metabolism studies (about 25~50 miligrams for these three compounds by p.o). In addition, the proposed zebrafish model is lower cost, easier to set up and has higher efficiency.

## 3. Experimental

### 3.1. Chemicals and Reagents

Tanshinone IIA and cryptotanshinone (purities were all >98%) were purchased from the National Institute for the Control of Pharmaceutical and Biological Products (Beijing, China), and tanshinone I (purity > 97%) was purchased from Sikehua Biotechnology Co., Ltd. (Chengdu, China). HPLC grade acetonitrile was purchased from Tedia Company (Fairfield, CT, USA), deionized water was purified by Milli-Q system (Millipore, Bedford, MA, USA), robust purified water, physiological saline (sodium chloride injection) were from Nanjing Xiaoying Pharmaceutical Group Co. Ltd. (Nanjing, China), dimethyl sulfoxide (DMSO) from Sinopharm Chemical Reagent Co. Ltd (Shanghai, China), the other reagents were analytical grade. 

### 3.2. Animals

The adult zebrafish (*D. rerio*) (about six month) of mixed sex were provided by Model Animal Research Center of Nanjing University (Nanjing, China), and acclimatized to tap water in a glass aquarium for at least 10 days prior to experimentation. Fish were kept at a temperature of 25 ± 1 °C in a photoperiod of 12:12 h. The fish were fed daily during the acclimatization period, and were fasted overnight before the day of the experiment.

### 3.3. Instruments

An Agilent Technologies modular 1200 system (Agilent Corporation, Santa Clara, CA, USA), equipped with a vacuum degasser, a binary pump, an autosampler, a thermostatted column compartment and a 6310A Ion Trap (IT) mass analyzer with an ESI ion source was used. Data were acquired by Agilent 6300 Series Ion Trap LC/MS system software (version 6.1); Mettler Toledo AB135-S Analytical Balance (Mettler Toledo, Schwerzenbach, Switzerland); KQ3200DE Digital Ultrasonic Washer (Kunshan Ultrasonic Instruments Co. Ltd., Kunshan, China); Labconcosk Centrifuge (Shanghai Anting Scientific Instrument Factory, Shanghai, China), Organomation N Freezer Dryer (Labconco, Kansas City, MO, USA); TGL-16G De -EVAP^TM^ 112 Nitrogen Evaporator (Organomation Associates, Inc., Berlin, MA, USA) were used too.

### 3.4. Biological Sample Collection

Adult zebrafish (about six month old) were divided into four experimental groups of five fish each. After fasting for 12 h, the five fishes in each group were kept individually in 60 mL glass bottles with 30 mL solution: one blank control group was exposed to 1% DMSO in purified water (blank zebrafish group), three groups were exposed to 30 mL solution of TIIA (1.78 µg/mL), Cry (1.72 µg/mL) and TI (1.88 µg/mL) in 1% DMSO purified water (drug zebrafish groups), respectively. In addition, the above solutions of TIIA (1.78 µg/mL), Cry (1.72 µg/mL) and TI (1.88 µg/mL) without zebrafish were used as blank drug controls. Both zebrafish body and solution of the blank zebrafish group and drug zebrafish groups were sampled at 24 h, respectively. the zebrafish bodys of each group were combined and washed rapidly with 1% DMSO purified water three times and stored at −70 °C prior to analysis; the solution of each group were also combined, respectively.8 mL (n = 3) combined solution of each group were sampled and also stored at −70 °C prior to analysis. Solution of blank drug control groups were sampled as above (8 mL, n = 3) at 0 h, 24 h.

### 3.5. Sample Preparation

The solution sample (8 mL) was freeze-dried to dryness, and the residue was dissolved in 90% methanol (1 mL). After centrifugation at 15,000 rpm for 15 min, supernatant (20 µL) was introduced into the HPLC-MS system for analysis. The zebrafish body samples (five fish of each group) were cut with scissors, and 1 g was sampled and homogenized with physiological saline (5 mL), followed by centrifugation at 3,500 rpm for 15 min, then the supernatant was extracted with ethyl acetate at the ratio of 1:3 (v/v) for three times, followed by centrifugation at 3,500 rpm for 10 min, the ethyl acetate fractions were affiliated and evaporated to dryness with nitrogen at room temperature, and the residue was dissolved in 90% methanol (1 mL). After centrifugation at 15,000 rpm for 10 min, 20 µL of the supernatant was injected into the HP LC-MS system for analysis.

### 3.6. Analysis Condition

HPLC/IT-MSn analysis was performed with an Agilent Technologies modular 1200 system combined with a 6310A Ion Trap (IT) mass analyzer with an ESI ion source. The IT parameters were set as follows: the capillary voltage was 3.5 kV, the nebulizer (N_2_) pressure was 35 psi, the drying gas (N_2_) temperature was 350 °C, the drying gas flow rate was 12.0 L/min. Data were acquired by Agilent 6300 Series Ion Trap LC/MS system software (version 6.1). IT was used in the full-scan positive ion mode in the *m/z* range 100–600. The [M+H]^+^ ion was selected as precursor ion and fragmented up to MSn stage. Chromatographic separation was performed on an Agilent Zorbax Extend reversed-phase C_18_ column (5 µm, 250 mm × 4.6 mm) and an Agilent Zorbax extend-C_18_ guard column (5 µm, 20 mm × 4 mm). A gradient elution of 0.05% aqueous formic acid (A) and 0.05% acetonitrile formic acid (B) was used as 45% B at 0~5 min, 45~50% B at 5~10 min, 50~90% B at 10~30 min, 90~100% B at 30~35 min. The column was maintained at 30 °C, the flow rate was 1.0 mL/min, and the LC effluent was introduced into the ESI source in a post-column splitting ratio of 2:1.

## 4. Conclusions

In conclusion, this study has confirmed that a zebrafish model can imitate regular methods in elucidating the phase I metabolism mechanism of TIIA, Cry and TI from Radix Salvia miltiorrhiza, which provide further useful evidence for the possibility and reasonability of using zebrafish in the metabolic study of compounds from TCMs. In comparison to the known metabolism, the zebrafish model possessed advantages of needing far less compound, lower cost, easier set up and higher efficiency in quickly predicating the *in vivo* metabolism of trace TCM components.
